# Simiao pill inhibits epithelial mesenchymal transition in a mouse model of chronic hyperuricemic nephropathy by inhibiting NLRP3 inflammasome activation

**DOI:** 10.1186/s12906-022-03757-0

**Published:** 2022-10-21

**Authors:** Guangxing Shui, Zheyi Cai, Feng Wang, Ting Chen, Xueyuan Huang, Yun Cai, Xiuhua Mi

**Affiliations:** 1 Department of Nephrology, Shanghai Jiading Hospital of Traditional Chinese Medicine, Shanghai, 201899 China; 2 Department of Second Clinical Medical School, Nanjing University of Chinese Medicine, Nanjing, 210029 China; 3 Department of Nephrology, Shanghai Baoshan Hospital of Integrated Traditional Chinese Medicine and Western Medicine, Shanghai, 201999 China

**Keywords:** Simiao pill, Epithelial-mesenchymal transition, NLRP3 inflammasome, Chronic hyperuricemic nephropathy

## Abstract

**Background:**

Simiao pill module (SMM), a traditional Chinese medicine formula, has been widely used to treat gout and gouty arthritis. The goal of this study was to investigate the effects of SMM on epithelial-mesenchymal transition (EMT) and activation of NLR family pyrin domain containing 3 (NLRP3) inflammasome in a mouse model of potassium oxonate (PO)-induced chronic hyperuricemic nephropathy (HN).

**Methods:**

Mice were randomly divided into the following four groups: control, HN model (PO), febuxostat (FEB)-treated (PO + FEB), and SMM-treated (PO + SMM) groups. Following 6 weeks of treatment, blood samples were collected and mice were sacrificed to collect kidney samples to study the biochemical parameters associated with renal function and histopathological changes associated with HN, respectively. The samples were analyzed for the expression of markers of EMT (collagen type 3, α-smooth muscle actin [α-SMA], fibronectin, vimentin and E-cadherin) and activation of NLRP3 inflammasome (NLRP3, apoptosis-associated speck-like protein [ASC], caspase-1, interleukin [IL]-1β, and IL-18).

**Results:**

Our results showed that hyperuricemia, impaired kidney function, and renal pathological characteristics induced by PO treatment were improved following treatment with SMM and FEB. Additionally, treatment with SMM and FEB decreased the expression of vimentin, collagen 3, fibronectin, and α-SMA, and increased the expression of E-cadherin. Moreover, NLRP3 inflammasome activation, as assessed by the increased expression of NLRP3, ASC, and caspase-1, and secretion of IL-1β and IL-18, was inhibited by treatment with SMM and FEB.

**Conclusion:**

These results suggest that SMM inhibited EMT and NLRP3 inflammasome activation in chronic HN mice, and the beneficial effect of SMM was compared with a standard drug, FEB.

**Supplementary Information:**

The online version contains supplementary material available at 10.1186/s12906-022-03757-0.

## Background

In recent years, hyperuricemia has become increasingly common worldwide, affecting approximately 13.3% of the population in China and up to 20.2% of the population in the United States [1]. Hyperuricemia is widely accepted to be associated with overproduction and underexcretion of uric acid (UA). The kidney and intestine play important roles in UA excretion, and more than 70% of UA is excreted through the kidney [2]. However, overproduction of UA such that it exceeds the excretion capacity of the kidneys or underexcretion of UA by the kidneys can result in kidney inflammatory injury, which further impairs renal functions and ultimately causes hyperuricemic nephropathy (HN) [3]. The incidence of chronic HN has increased significantly in the past decades and is associated with high morbidity and mortality. Therefore, the mechanisms underlying the pathogenesis of chronic HN should be explored further.

Tubular-interstitial fibrosis is a characteristic feature of chronic HN, and growing evidence suggests that epithelial-mesenchymal transition (EMT) plays an important role in the progression of tubulointerstitial renal fibrosis [4]. Epithelial cells undergo gradual biochemical changes and transition to mesenchymal-like cells, losing their epithelial characteristics during EMT. High UA levels were reported to induce EMT in tubular cells of hyperuricemic rats’ kidneys by increasing the expression of α-smooth muscle actin (α-SMA) and decreasing the expression of E-cadherin [5]. Liu et al. demonstrated that high UA levels could induce EMT in human renal tubular epithelial cells by activating the TLR4/NF-κB signaling pathway. Another study reported that high UA levels activate the PI3K/p-Akt signaling pathway and induce EMT in renal tubular cells [6, 7]. Although several signaling pathways have been reported, further studies are required to elucidate the underlying mechanisms of hyperuricemia-induced EMT in tubular epithelial cells.

The NLR family pyrin domain-containing 3 (NLRP3) inflammasome is a multiprotein complex composed of NLRP3, apoptosis-associated speck-like protein (ASC), and caspase-1. High UA levels trigger the formation of the NLRP3 inflammasome, which mediates the secretion of interleukin (IL)-1β and IL-18 and plays a crucial role in renal inflammation [8]. Some studies have demonstrated that IL-1β can enhance the activity of transforming growth factor (TGF)-β, which consequently induces EMT [9]. Romero et al. found that there is a physical interaction between NLRP3 and the transcription factor Smad2/3, which regulates the expression of genes responsible for EMT [10]. Song et al. reported that silencing of NLRP3 in high glucose-induced renal tubular fibrosis results in reduced TGF-β levels and inhibition of EMT [11]. The findings of these studies suggest that the NLRP3 inflammasome is an upstream signaling pathway in EMT. Therefore, inhibiting the activation of NLRP3 inflammasome may provide a potential therapeutic strategy to improve renal inflammation and fibrosis.

The Simiao pill module (SMM), which consists of *Atractylodes lancea* (Thunb.) DC., *Phellodendron amurense* Rupr., *Coix lacryma-jobi* L., and *Achyranthes bidentata* Blume, is a famous traditional Chinese medicine formula that has been used for hundreds of years. It has been widely reported to be effective and safe in treating gout and gouty arthritis in patients [12–14]. In a hyperuricemic mouse model induced by potassium oxonate (PO) and yeast polysaccharide, hyperuricemia was inhibited after treated with SMM [15]. Lin et al. found that SMM was effective for reducing the level of serum UA and suppressing NLRP3 inflammasomes expression in gouty arthritis mouse [16]. Another research observed that SMM ameliorated renal glomerular injury via suppressing NF-κB /NLRP3 inflammasome activation in high fructose-fed rats [17]. All these evidence showed SMM has potential in lowering level of serum UA, inhibiting NLRP3 inflammasome activation and improving renal damage. Thus, the effects of SMM on the EMT and NLRP3 inflammasome activation in chronic HN which was caused by chronic hyperuricemia deserve well study [18–20]. In the present study, we developed a mouse model for chronic HN by intraperitoneal administration of PO to further investigate the effects of SMM on renal function, EMT, and NLRP3 inflammasome activation.

## Methods

### Reagents

PO (156124) and febuxostat (FEB) (SML1285) were purchased from Sigma–Aldrich (St. Louis, MO, USA). The primary antibodies used in the present study were purchased from Bioworld Technology (Nanjing, China) and were as follows: rabbit anti-collagen 3A1, rabbit anti-α-SMA, rabbit anti-fibronectin, rabbit anti-vimentin, rabbit anti-E-cadherin, rabbit anti-NLRP3, rabbit anti-ASC, rabbit anti-caspase-1, rabbit anti-IL-1β, rabbit anti-glyceraldehyde 3-phosphate dehydrogenase (GAPDH), and rabbit anti-TGFβ1 (BS7028, BS70000, BS90514, BS1491, BS1098, BS90948, BS6205, BS5641, AP0063, BS72410,BS91338). The assay kits for UA (ab65344), creatinine (ab65340), xanthine oxidase (XOD) (ab102522), IL-1β (ab197742), and IL-18 (ab216165) were purchased from Abcam (Cambridge, UK). The PrimeScript™ RT Master mix was obtained from Takara (Takara, Japan) and the FastStart Universal SYBR Green master mix was obtained from Roche (Basel, Switzerland).

### Preparation of SMM

SMM was prepared using the method described by Shui et al. (2017) [21]. The SMM granules contained 9 g of *A, lancea* (Thunb.) DC. (no. 1201076), 3 g of *P. amurense* Rupr. (no. 1210012), 9 g of *C. lacryma-jobi* L. (no. 1210132), and 4 g of *A. bidentata* Blume (no.1208203), which were purchased from Tianjiang Pharmacology Co. Ltd. (Jiangyin, China). Herb granules (a total weight of 25 g) were dissolved in 25 mL saline for a final concentration of 1 g/mL.

### Animals and drug treatment

All experiments involving animals were carried out according to the guideline for the care and use of laboratory animals and approved by the Animal Ethics Committee of Nanjing University of Chinese Medicine (approval number: ACU210306, Nanjing, China). This study is reported in accordance with ARRIVE guidelines (https://arriveguidelines.org). Eight week old C57BL/6 background male mice were purchased from the Experimental Laboratory of Animal Models (Nanjing, China) and housed at the Experimental Animal Center of Nanjing University of Chinese Medicine. Mice were randomly divided into four groups with eight mice in each group. The control group received intraperitoneal saline injections and saline by oral gavage. The PO group received intraperitoneal injections of 300 mg/kg PO daily for 6 weeks [22]. Three weeks into PO treatment, the PO + FEB group received 5.2 mg/kg of FEB daily by oral gavage for 21 days. Similarly, the SMM-treated group (PO + SMM) received 350 mg/kg of SMM daily once by oral gavage for 21 days. At the end of the 6 weeks treatment period, the mice were anaesthetised with 3% pentobarbital sodium at dosage of 40 mg/kg through intraperitoneal injection and sacrificed using cervical dislocation. Blood samples were obtained from the eye socket vein of each mouse. The kidney tissues were collected immediately for further examination.

### Renal function tests and levels of inflammatory cytokines

Serum UA, serum blood urea nitrogen, serum creatinine, IL-1β, and IL-18 levels were measured using assay kits (Abcam) according to the manufacturer’s instructions.

### Periodic acid–Schiff staining, Masson’s trichrome staining, and immunohistochemistry of kidney tissue samples

Kidney samples were fixed with 4% paraformaldehyde, embedded in paraffin, and sectioned transversely. Kidney sections (2 μm) were stained with Periodic acid–Schiff and Masson’s trichrome. Pathological changes were observed under a light microscope and images were captured. Renal histological damage was evaluated using semi quantitative scoring. For immunohistochemical staining, the sections were blocked with 10% goat serum and incubated with IL-1β antibody at 4 °C overnight, followed by incubation with a horseradish peroxidase (HRP)-conjugated secondary antibody for 1 h at room temperature. Then, the sections were incubated with streptavidin-HRP (IHC003, Bioss, Beijing, China). Images were captured using light microscope and analyzed using the ImageJ software (National Institutes of Health, Bethesda, MD, USA) to assess IL-1β expression in the kidney.

### Immunofluorescence staining

Kidney sections (2 μm) were deparaffinized and blocked with 10% goat serum at 37 °Cfor 30 min. The sections were then incubated with primary antibodies against α-SMA and fibronectin at 37 °C for 4 h and visualized using a secondary antibody. The nuclei were counterstained with 4′,6-diamidino-2-phenylindole. Images were captured using a fluorescence microscope.

### Western blot analysis

The expression profiles of collagen 3, α-SMA, fibronectin, vimentin, E-cadherin, and NLRP3 inflammasomes were analyzed by western blot analysis as we described previously [21]. Blots were visualized using the Amersham ECL Detection System (Cytiva, Marlborough, MA, USA). ImageJ software was used to quantify the band intensities normalized to the expression of GAPDH, the loading control.

### Quantitative real time PCR (qRT-PCR)

Target and reference genes were detected using real-time PCR [21]. The primer pair sequences were as follows: NLRP3, forward: 5′-GTGGTGACCCTCTGTGAGGT-3′, reverse: 5′ -TCTTCCTGGAGCGCTTCTAA-3′; ASC, forward: 5′-AGACATGGGCTTACAGGA-3′, reverse: 5′-CTCCCTCATCTTGTCTTGG-3′; caspase-1, forward: 5′-TATCCAGGAGGGAATATGTG-3′, reverse: 5′-ACAACACCACTCCTTGTTTC-3′; IL-1β, forward: 5′-GCCCATCCTCTGTGACTCA-3′, reverse: 5′-AGTTGTCTGATTCCAGGTCTCCAT-3′; GAPDH, forward: 5′-GGAGCGAGATCCCTCCAAAAT-3′, reverse: 5′-GGCTGTTGTCATACTTCTCATGG-3′.

### Statistical analysis

All data were analyzed using SPSS version 21.0 (IBM, Armonk, NY, USA) and presented as the mean ± standard deviation. Student’s t-test was used for comparisons between two groups. Comparisons between multiple groups were analyzed using ANOVA, and a nonparametric test was used when necessary. Statistical significance was set at *P* < 0.05.

## Results

### SMM inhibited XOD activity in liver, decreased the UA level and improved renal function in the chronic HN model

**Fig. 1:**
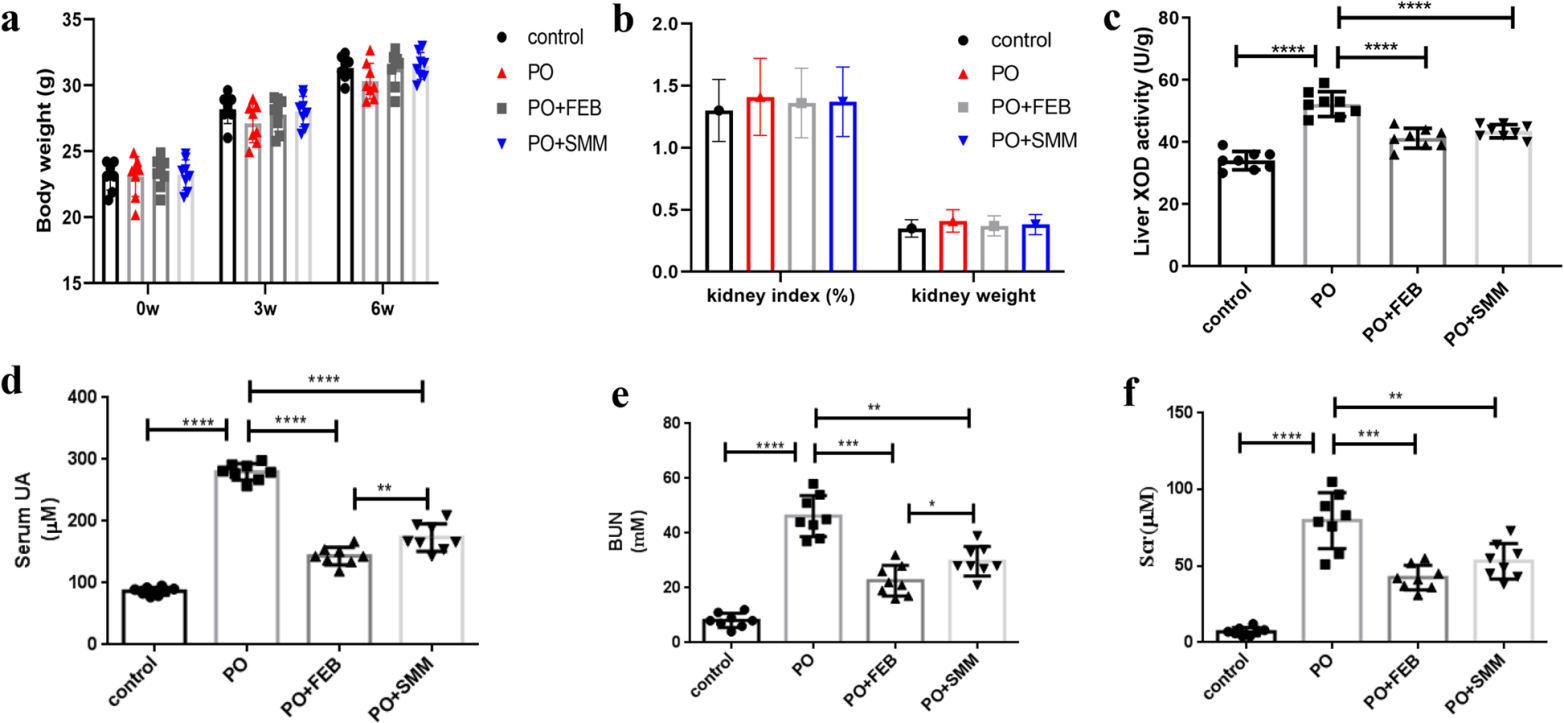
Effects of Simiao pill module (SMM) on **a** body weight (BW), **b** kidney weight (KW) and kidney index (KW/BW), **c** liver xanthine oxidase (XOD) activity, **d** serum uric acid (UA), **e** blood urea nitrogen (BUN), and **f** serum creatinine (Scr) in chronic hyperuricemic nephropathy (HN mice (*n* = 8). SMM improved **a** the loss of BW, and **b** increase in KW and kidney index induced by potassium oxonate (PO) injection. **c** SMM inhibited the activity of liver XOD. SMM decreased **c** UA levels and **e** and **f** ameliorated renal function as assessed by **e** BUN and **f** Scr.*p < 0.05, **p < 0.005, ***P < 0.001, ****P < 0.0001

We investigated whether SMM treatment improved renal function in a mouse model of chronic HN and also evaluated the effect of FEB on the same. The PO + SMM and PO + FEB groups were also evaluated. Two physical and three nephropathy-related biochemical parameters were used to determine disease severity. As shown in Fig. 1, PO injection resulted in the loss of overall body weight (BW) and an increase in kidney weight (KW) and kidney index (KW/BW) compared to the control group. In addition, PO treatment result in a considerable increase in liver XOD activity, high serum UA levels, and impairment of kidney function, which was characterized by elevated levels of blood urea nitrogen and serum creatinine compared to the control group. As expected, significant improvements in BW, KW, kidney index, liver XOD activity, and other kidney function parameters were observed in the PO + SMM and PO + FEB groups (Fig. 1).

### SMM improved histopathological changes in the kidneys in the chronic HN model

**Fig. 2:**
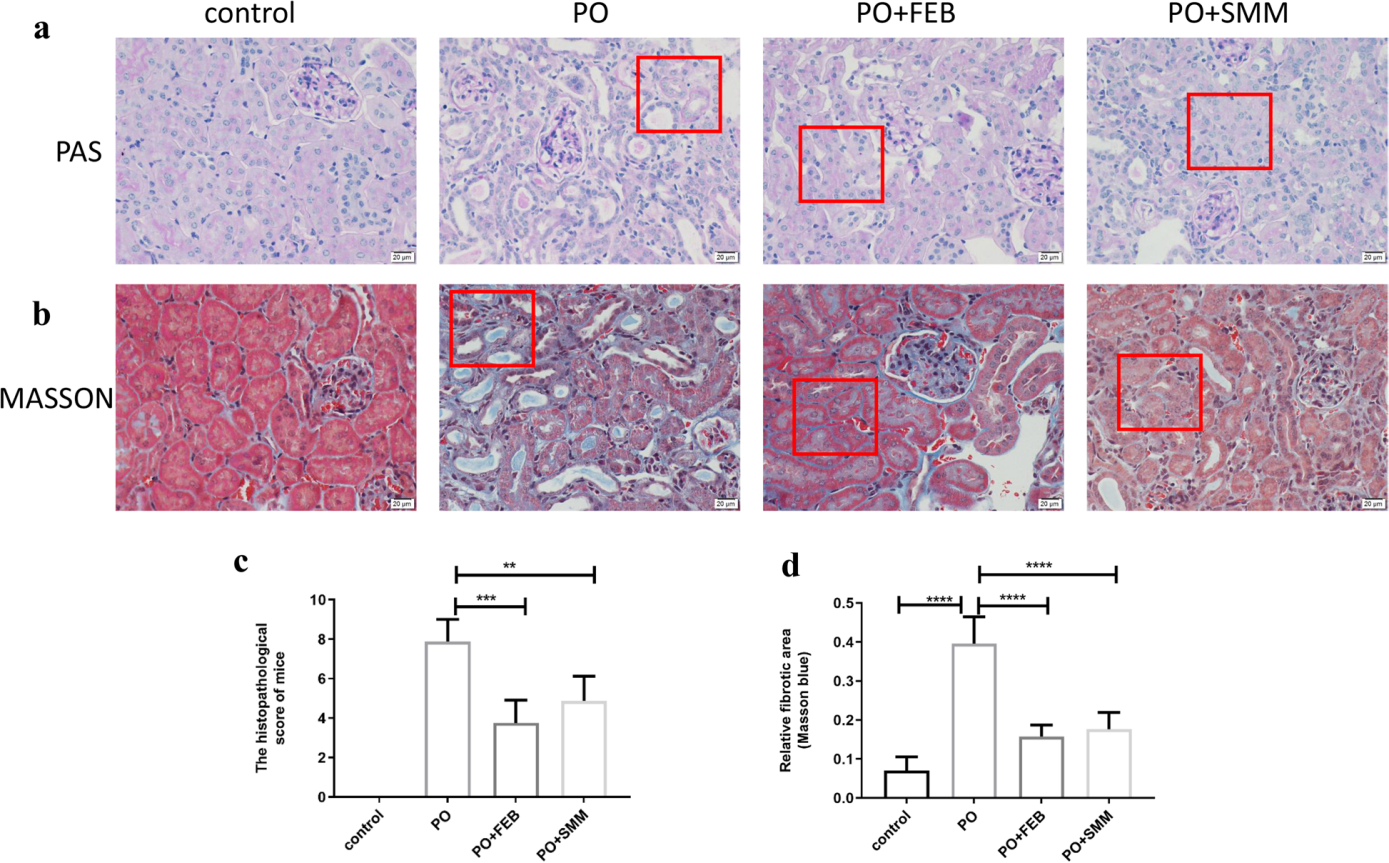
Effects of SMM on histopathological changes in chronic HN mice. Representative images of **a** Periodic acid–Schiff (400×) and **b** Masson trichrome staining (400×) to assess kidney histopathology. SMM decreased (**c**) histological severity scores and SMM improved histopathological changes in the kidneys in the chronic HN model (**d**) renal fibrosis in chronic HN mice (*n* = 8). **p < 0.005, ***P < 0.001, ****P < 0.0001

Renal histology revealed glomerular sclerosis, tubular dilatation, and dark-stained cell infiltration in kidney tissues from chronic HN mice (Fig. 2a). The level of cell infiltration and tissue damage was used for determining histological severity scores (Fig. 2c). Both SMM and FEB considerably improved the histopathological changes observed in chronic HN mice. Furthermore, blue-stained collagen was observed only around the basement membranes and blood vessels in normal kidney tissues (Fig. 2b). However, in chronic HN tissues, excessive deposition of collagen was observed around the kidney tissue, suggesting an increase in fibroblast proliferation and collagen secretion (Fig. 2b). Treatment with SMM and FEB resulted in a considerable decrease in collagen deposition (Fig. 2d).

### SMM inhibits EMT in tubular epithelial cells in the chronic HN model

**Fig. 3:**
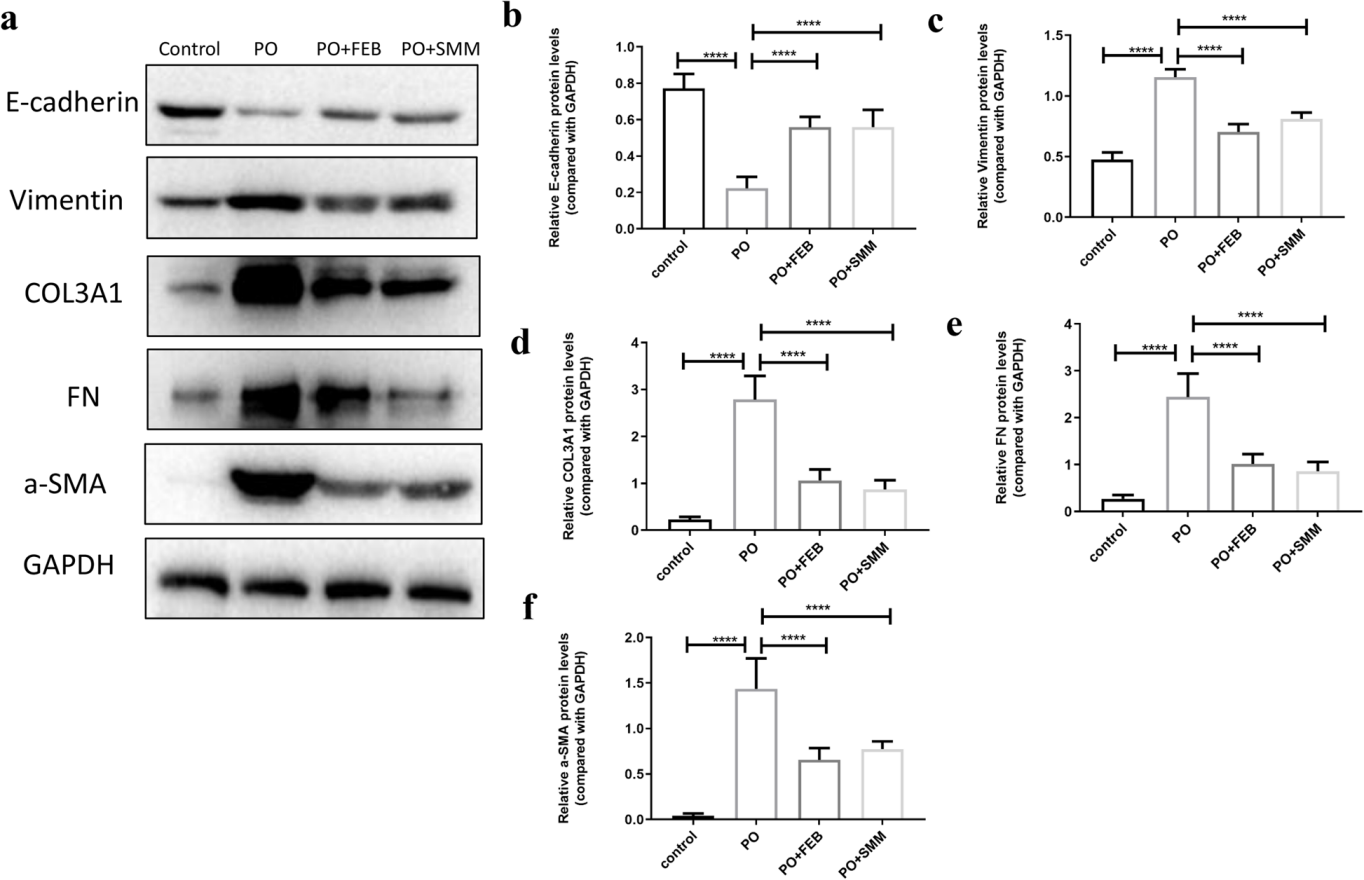
Western blot analysis for the expression of E-cadherin, vimentin, collagen 3, fibronectin and α-SMA in renal tissues. **a** Representative immunoblots of protein expression in the control, PO, PO + FEB, and PO + SMM groups. Densitometry analysis for western blot results of **b** E-cadherin, **c** vimentin, **d** collagen 3, **e** fibronectin, and **f** α-SMA protein (*n* = 3). ****P < 0.0001

**Fig. 4:**
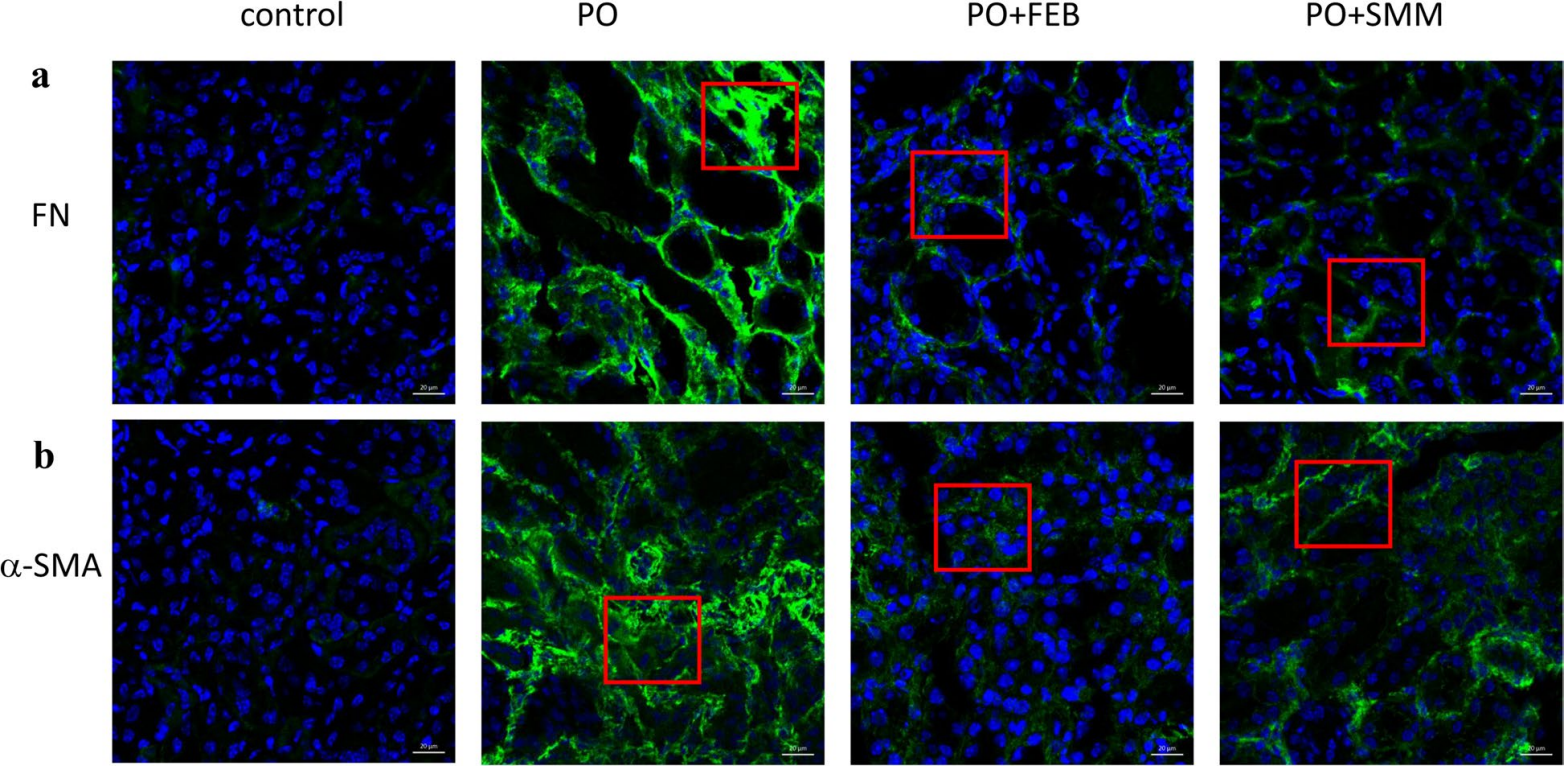
Representative images of immunofluorescence staining (400×) for the expression of fibronectin and α-SMA in renal tissues. SMM decreased the expression of **a** fibronectin and **b** α-SMA in PO-induced chronic HN mice

To confirm the effects of SMM on EMT in tubular epithelial cells in chronic HN mice, western blot analysis and immunofluorescence staining were performed. We observed that in kidney tissues from the HN group, protein levels of vimentin, collagen 3, fibronectin, and α-SMA were increased, while protein levels of E-cadherin were decreased in comparison to that in the control group (Fig. 3a). While in the PO + SMM and PO + FEB groups, the expression of vimentin, collagen 3, fibronectin, and α-SMA was inhibited, whereas the expression of E-cadherin was upregulated (Fig. 3a). Figures 3b–f show quantitative analysis of protein expression relative to GAPDH. Additionally, immunofluorescence staining confirmed that SMM and FEB treatment decreased the expression of fibronectin and α-SMA (Fig. 4a and b). These findings are consistent with the changes observed in histopathological examination, suggesting that SMM could protect against PO-induced chronic HN by reversing EMT in tubular epithelial cells.

### SMM inhibited PO-induced activation of the NLRP3 inflammasome and secretion of inflammatory cytokines in the chronic HN model

**Fig. 5:**
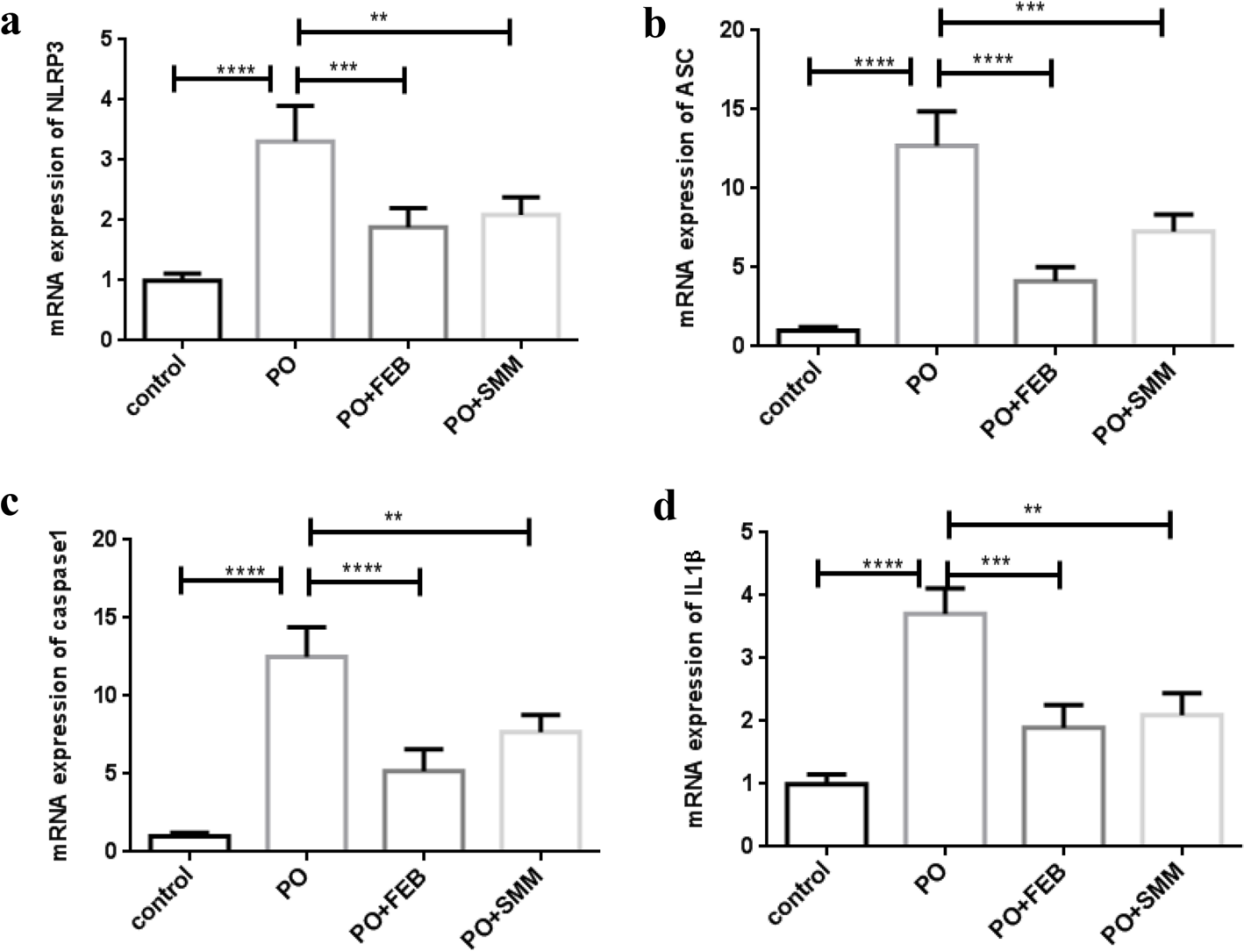
Relative gene expression levels of **a** NLR family pyrin domain containing 3 (NLRP3), **b** apoptosis-associated speck-like (ASC), **c** caspase-1, and **d** IL-1β in the different groups (*n* = 8). **p < 0.005, ***P < 0.001, ****P < 0.0001

**Fig. 6:**
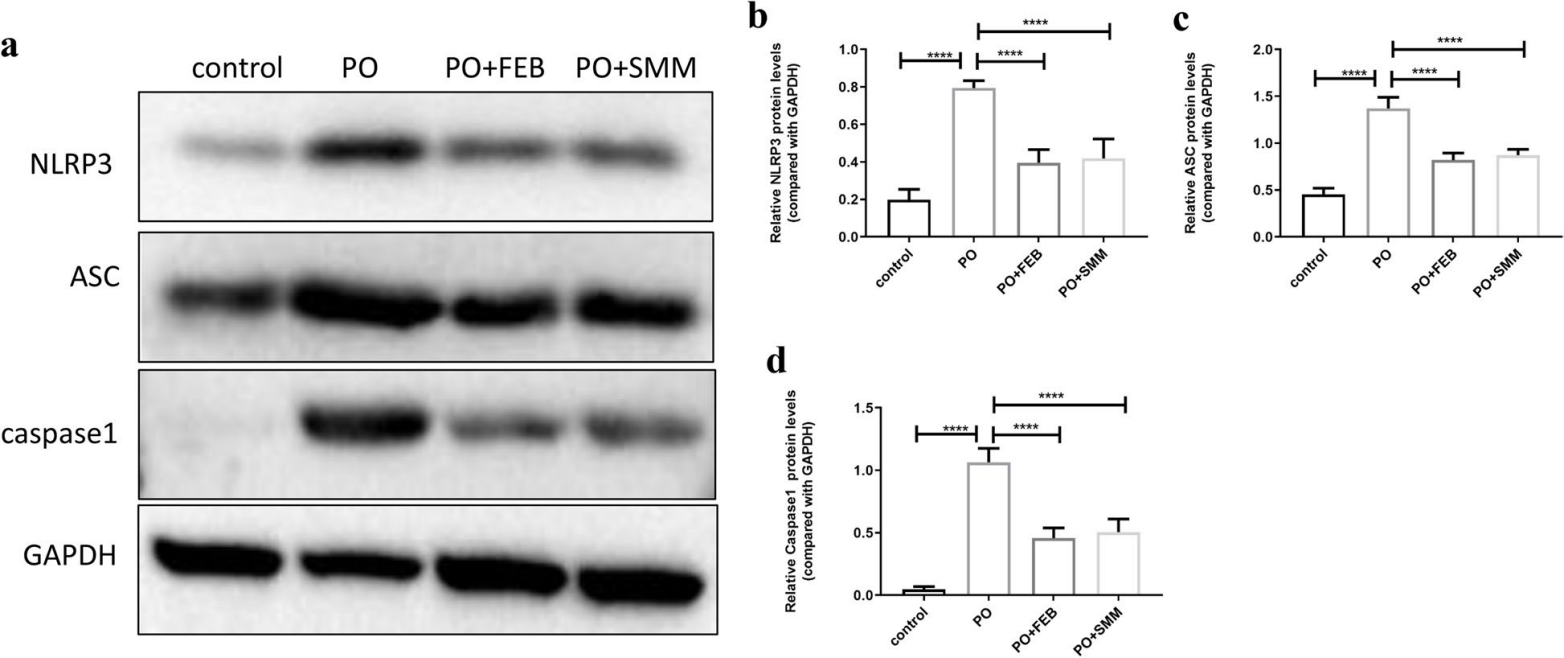
Western blot analysis for the expression of NLRP3, ASC and caspase-1 in renal tissues. **a** Representative immunoblots of protein expression in the control, PO, PO + FEB, and PO + SMM groups. Densitometry analysis for western blot results of **b** NLRP3, **c** ASC, and **d** caspase-1 (*n* = 3). ****P < 0.0001

**Fig. 7:**
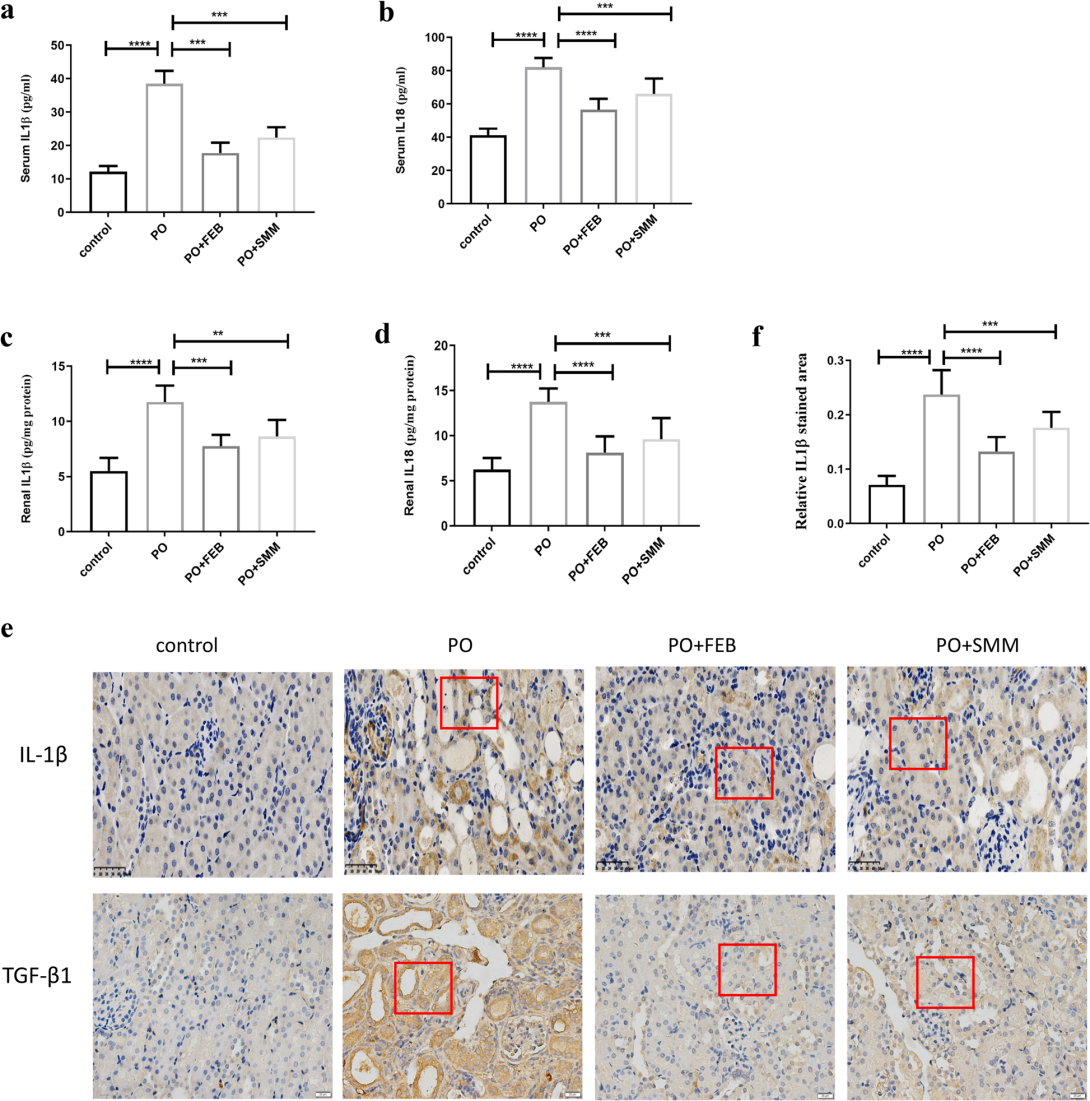
The secretion levels of IL-1β and IL-18 in the control, PO, PO + FEB, and PO + SMM groups. The serum levels of **a** IL-1β and **b** IL-18 in the different groups (*n* = 8). The protein levels of **c** IL-1β and **d** IL-18 in kidney tissues lysates (*n* = 8). **e** Representative images for immunohistochemical staining (400×) for the expression of IL-1β and TGFβ1 in renal tissues. **f** Relative IL-1β staining area in renal tissues (*n* = 3). **p < 0.005, ***P < 0.001, ****P < 0.0001

Previous studies have shown that activation of the NLRP3 inflammasome is involved in the progression of EMT [10]. Therefore, we investigated whether SMM inhibited the activation of NLRP3 inflammasome in chronic HN mice. To this effect, the relative gene and protein expression levels of components of the NLRP3 inflammasome were analyzed. In addition, assay kits and immunohistochemistry were used to analyze IL-1β and IL-18 levels, as the expression of these cytokines reflects activation of the NLRP3 inflammasome. As expected, PO treatment resulted in considerable activation of the NLRP3 inflammasome in kidneys from chronic HN mice compared to the control group (Figs. 5, 6 and 7). However, this effect was considerably inhibited by treatment with SMM and FEB (Figs. 5, 6 and 7). The relative gene expression levels of NLRP3, ASC, caspase-1, and IL-1β are shown in Fig. 5a-d. Representative immunoblots and densitometry analysis of the protein levels of NLRP3, ASC, and caspase-1 are shown in Fig. 6a-d. Consistent with the above results, increased secretion of IL-1β and IL-18 observed in chronic HN mice was considerably inhibited by treatment with SMM and FEB (Fig. 7a-d). Immunohistochemistry further confirmed that IL-1β and TGFβ1 secretion in the kidney was inhibited by treatment with SMM and FEB (Fig. 7e-f).

## Discussion

Chronic HN caused by chronic hyperuricemia can lead to renal damage and subsequently end-stage renal disease (ESRD) and has become increasingly more common worldwide. In the past decades, reducing serum UA levels has been the major treatment strategy for chronic HN. However, some clinical studies have found that although the levels of serum UA decrease with treatment with FEB or allopurinol, the progression of chronic HN to ESRD was not affected [23, 24]. Hence, it is necessary to find complementary strategies to treat chronic HN and delay its progression to ESRD. SMM is a traditional Chinese formula widely used to treat gout and gouty arthritis. SMM has been reported to lower serum UA levels and improve symptoms of gouty arthritis [16]. In the present study, we demonstrated that oral administration of SMM at a dose of 350 mg/kg/d significantly decreased the levels of serum UA, BUN, and Scr, suggesting that SMM can improve renal function in chronic HN mice and may have the potential to delay the progression of chronic HN to ESRD.

XOD plays a critical role in the conversion of xanthine and hypoxanthine to UA, higher XOD activity can lead to over synthesis of UA [25]. Some reports observed XOD level in liver was increased in PO-induced HN model mice which was consistent with our findings [22, 26]. Treatment of SMM with dosage of 350 mg/kg/d could significantly inhibit the activity of XOD in liver, indicating that SMM might decrease the level of UA through inhibiting XOD activity.

Hyperuricemia-induced EMT has been implicated in the initiation of renal fibrosis, which is one of the main characteristics of chronic HN. Kang et al. reported that UA-induced EMT in cultured renal tubular cells resulted in increased expression of Snail and decreased expression of E-cadherin [27]. Romi et al. reported that hyperuricemia was associated with EMT prior to the development of tubulointerstitial fibrosis [28]. Therefore, targeting EMT in tubular epithelial cells may provide novel strategies to improve renal function in chronic HN. We observed that SMM improved histopathological changes in renal fibrosis, downregulated the expression of vimentin, collagen 3, fibronectin and α-SMA, and upregulated the expression of E-cadherin. These results suggest that SMM inhibited EMT in tubular epithelial cells in chronic HN mice.

Several signaling pathways have been reported to be involved in the mechanisms underlying hyperuricemia-induced EMT. Among these, NLRP3 inflammasome activation has been reported. As a damage-associated molecular pattern, UA increases the expression of IL-1β and IL-18 by activating the NLRP3 inflammasome inflammatory pathway [29]. Romero et al. observed that NLRP3/ASC expression increased in tubular epithelial cells of rats with high UA levels, and inflammasome-related caspase-1 was associated with EMT [10]. Other studies have reported that the NLRP3 inflammasome is associated with myofibroblast differentiation during renal fibrosis [28, 30]. In the present study, we observed that SMM decreased the expression of NLRP3, ASC, and caspase-1, and secretion of IL-1β and IL-18. TGFβ1 is an important mediator involved in the progress of EMT. Wang et al. reported that silencing IL-1β decreased TGFβ1 expression and alleviated EMT in obstructive kidney diseases [31]. Other studies demonstrated IL-1β can enhance the activity of TGFβ1 and aggravate EMT [9]. We observed that administration of SMM decreased the secretion of IL-1β and TGFβ1, which indicated that SMM may inhibit EMT through NLRP3/ IL-1β/ TGFβ1 signaling. Taken together, these results suggest that SMM may inhibit EMT in kidneys of chronic HN mice by inhibiting the activation of NLRP3 inflammasome.

## Conclusion

In the present study, we identified the beneficial effect of SMM in treating chronic HN compared with a standard drug, FEB. The results demonstrated that SMM could decrease the level of UA, improve kidney function and protect against renal fibrosis by inhibiting EMT and activation of NLRP3 inflammasome in tubular epithelial cells of chronic HN mice model induced by PO.

## Supplementary Information

**Additional file 1.**

**Additional file 2.**

## Data Availability

All data generated or analysed during this study are included in this published article and its supplementary information files.

## Abbreviations

HN: hyperuricemic nephropathy
BUN: blood urea nitrogen
FEB: febuxostat
PAS: periodic acid–Schiff
PO: potassium oxonate
Scr: serum creatinine
SMM: Simiao pill module
UA: uric acid
XOD: xanthine oxidase
FN: fibronectin
GAPDH: glyceraldehyde 3-phosphate dehydrogenase
α-SMA: α- smooth muscle actin
ASC: apoptosis-associated speck-like
NLRP3: NLR family pyrin domain containing 3
IL: interleukin
