# Supplementary material for: Simiao pill inhibits epithelial mesenchymal transition in a mouse model of chronic hyperuricemic nephropathy by inhibiting NLRP3 inflammasome activation
Source: BMC Complement Med Ther. 2022 Oct 21;22:278. doi: 10.1186/s12906-022-03757-0 (PMC9587568; doi:10.1186/s12906-022-03757-0)
Supplement: Supplementary file 1 — Additional file 1. [file 12906_2022_3757_MOESM1_ESM.docx]

Qualitative analysis of SMM

The quality stability and repeatability of SMM were detected by UPLC-MS/MS analysis method. More than forty compounds were identified in SMM which was consistent with Lu’s report [1] (Table S1).

Chromatographic separation was performed on an Agilent 1290 series UHPLC system (Agilent Technologies, CA, USA) with Agilent Poroshell SB-C18 column (100mm × 2.1mm, 2.7μm). The mobile phase was consisted of 0.1% formic acid in water (A) and 0.1% formic acid in acetonitrile (B). The gradient elution was carried out as follows: 0−16.7min, 10%−25% B; 16.7−20min, 25%−40% B; 20−30min, 40%−70% B; 30−33.3min, 70%−95% B; and 33.3−36.7min, 95% B. The flow rate was set at 0.2mL·min^−1^, the injection volume was set at 2μL, and the column temperature was maintained at 25°C. The LC system was coupled to a 6546 Q-TOF Mass Spectrometer (Agilent Corp., Santa Clara, CA, USA) equipped with an electrospray ionization (ESI) source. The conditions of the MS system were set as follows: drying gas temperature, 325°C; drying gas flow rate, 8.0 L·min^−1^; nebulizer gas pressure, 35 psi; sheath gas temperature, 350°C; sheath gas flow rate, 11.0 L·min^−1^; capillary voltage, 3500V; and fragmentor voltage, 120V. Analysis using an auto-MSMS mode with two collision energies (15 and 30V) was performed in positive and negative modes and the masses were scanned from 50 to 1, 100 Da without any preferred ion list. The instrument was working in the 10GHz high-resolution mode, with the acquisition rates being 1 spectrum/s for MS data and 2 spectras for the MS/MS data. Nitrogen was used as the drying and collision gas. To increase mass accuracy, the Q-TOF mass spectrometer was calibrated before analysis and subsequently using the internal reference masses at m/z 121.050 8 and m/z 922.009 8 in the positive ion mode, and at m/z 112.988 5 and m/z 1 033.988 1 in the negative ion mode. The procedures of acquisition were operated under Agilent MassHunter Data Acquisition Software Version 10.1(Agilent Technologies). The raw data (chromatograms) were processed using the Molecular Feature Extractor algorithm within Agilent MassHunter Qualitative Analysis Software Version 10.0 (Agilent Technologies) (Figure S1).

Table S1. Compounds identified in SMM

| No | Identification | No | Identification |
| --- | --- | --- | --- |
| 1 | Valine | 22 | Obaculactone |
| 2 | Candicine | 23 | Atractylenolide Ⅰ |
| 3 | Oblongine | 24 | AtractylenolideⅢ |
| 4 | Phellodendrine | 25 | Obacunone |
| 5 | Tembetarine | 26 | AtractylenolideⅡ |
| 6 | Magnoflorine | 27 | 4-Boc-piperazine-2-carboxylic acid |
| 7 | Lotusine | 28 | Selina-4(14),7(11)-dien-8-one |
| 8 | N-Methyltetrahydrocolumbamine | 29 | Tetramethy1-O-Scutellarin |
| 9 | Ecdysterone | 30 | Quinic acid |
| 10 | Menisperine | 31 | Malic acid |
| 11 | Inokosterone | 32 | Neochlorogenic acid |
| 12 | Demethyleneberberine | 33 | Chlorogenic acid |
| 13 | Tetrahydropalmatine | 34 | Cryptochlorogenin acid |
| 14 | Oxyberberine | 35 | Caffeoyl-CH_2_-O-quinic acid |
| 15 | Columbamine | 36 | 3-O-Feruloylquinic acid |
| 16 | Jatrorrhizine | 37 | Ferulic acid |
| 17 | Thalifendine | 38 | Amurenlaetone B |
| 18 | Berberrubine | 39 | Nonanedioic acid |
| 19 | N-Methyl canadine | 40 | Sanleng acid |
| 20 | Palmatine | 41 | Galnon |
| 21 | Berberine |  |  |

Positive ion

Negative ion

Figure S1. The representative total ion chromatograms (TICs) of SMM in positive and negative ion modes

References

1. J. J. Lu, X. W. Hu, P. Li, J. Chen. Global identification of chemical constituents and rat metabolites of Si-Miao-Wan by liquid chromatography-electrospray ionization/quadrupole time-of-flight mass spectrometry. Chin J Nat Med, 2017; 15:550-560.
